# Effects of Sodium–Glucose Cotransporter 2 Inhibitors in Diabetic and Non-Diabetic Patients with Advanced Chronic Kidney Disease in Peritoneal Dialysis on Residual Kidney Function: In Real-World Data

**DOI:** 10.3390/medicina60081198

**Published:** 2024-07-24

**Authors:** Esperanza Moral Berrio, José C. De La Flor, Minerva Arambarri Segura, Pablo Rodríguez-Doyágüez, Alberto Martínez Calero, Rocío Zamora, Michael Cieza-Terrones, Claudia Yuste-Lozano, María Dolores Sánchez de la Nieta García, Javier Nieto Iglesias, Carmen Vozmediano Poyatos

**Affiliations:** 1 Department of Nephrology, Hospital General Universitario de Ciudad Real, 13005 Ciudad Real, Spain; emoral@sescam.jccm.es (E.M.B.); marambarri@sescam.jccm.es (M.A.S.); amcalero@sescam.jccm.es (A.M.C.); mdoloressd@sescam.jccm.es (M.D.S.d.l.N.G.); ljnietoi@sescam.jccm.es (J.N.I.); mcarmenv@sescam.jccm.es (C.V.P.); 2 Department of Nephrology, Hospital Central de la Defensa Gómez Ulla, 28047 Madrid, Spain; 3 Department of Medicine and Medical Specialties, Faculty of Medicine, Alcala University, 28805 Madrid, Spain; 4 Department of Nephrology, Guadalajara Center Dialysis, AVERICUM, 19003 Guadalajara, Spain; pablo.rodriguez@avericum.com; 5 Department of Nephrology, Hospital Universitario General Villalba, 28400 Madrid, Spain; rocio.zamora@quironsalud.es; 6 Faculty of Medicine, Peruana Cayetano Heredia University, Lima 15002, Peru; michael.cieza@upch.pe; 7 Department of Nephrology, Hospital 12 de Octubre, 28041 Madrid, Spain; claudia.yuste@salud.madrid.org

**Keywords:** sodium–glucose cotransporter 2 inhibitors, peritoneal dialysis, residual kidney function

## Abstract

*Background and Objectives*: Peritoneal dialysis (PD) is a renal replacement therapy modality in which the dialysis dose can be individually adapted according to the patients’ residual kidney function (RKF). RKF is a crucial factor for technique and patient survival. Pharmacological strategies aimed at slowing the loss of RKF in patients on PD are limited. Therefore, we aimed to assess the potential effects and safety of sodium–glucose cotransporter 2 (SGLT-2) inhibitors on the preservation of RKF in patients with and without type 2 diabetes mellitus (T2DM) on PD during an average follow-up of 6 months. *Materials and Methods*: In this retrospective observational, single-center study on real-world data, we included patients from the Peritoneal Dialysis Unit of the Hospital General Universitario de Ciudad Real, who started treatment with SGLT-2 inhibitors during the period from December 2022 to December 2023. Data on analytical and clinical parameters, RKF, and peritoneal membrane transport function were retrospectively collected at months 0, 3, and 6. *Results*: Out of 31 patients in our unit, 16 prevalent patients initiated treatment with SGLT-2 inhibitors (13 empagliflozin and 3 dapagliflozin). A total of 62.5% were male and the mean age was 67.3 years. The baseline peritoneal ultrafiltration was higher in the non-diabetic patient (NDMP) group than in the diabetic patient (DMP) group. However, the residual diuresis volume, 24 h residual renal clearance rate of urea in urine, and 24 h proteinuria were higher in the DMP group than in the NDMP group. At the sixth month, patients in both groups preserved RKF and diuresis, with a trend towards a non-significant reduction in proteinuria and blood pressure. Only two patients of the DMP group presented adverse effects. *Conclusions*: The use of SGLT-2 inhibitors in our sample of patients with and without T2DM on PD appears to be safe and effective to preserve RKF.

## 1. Introduction

There has been an evolution in clinical guidelines over the past decade regarding the indications for the treatment of chronic kidney disease (CKD) and type 2 diabetes mellitus (T2DM) with sodium–glucose cotransporter 2 (SGLT-2) inhibitors. Initially approved as hypoglycemic agents, they have now emerged as one of the cornerstones in the treatment of CKD in patients with and without T2DM, thanks to the renal and cardiovascular benefits demonstrated in various clinical trials [1,2,3]. Additionally, renal outcomes from randomized clinical trials (RCTs) have demonstrated other benefits of SGLT-2 inhibitors, such as delayed progression to end-stage kidney disease (ESKD), reduced glomerular hyperfiltration and proteinuria, and improved cardiovascular outcomes and all-cause mortality [1,2,4], even in patients with advanced chronic kidney disease (ACKD) with and without T2DM going down to an estimated glomerular filtration rate (eGFR) of 20 mL/min/per 1.73 m^2^ [3]. Another cardiovascular benefit is their effect in reducing the incidence of atrial fibrillation [5]. It is still unclear whether these effects extend to populations on renal replacement therapy (RRT), such as hemodialysis (HD) or peritoneal dialysis (PD).

Given that SGLT-2 inhibitors act by inhibiting the reabsorption of sodium and glucose in the proximal convoluted tubule, the glucosuric efficacy of SGLT-2 inhibitors diminishes in patients with eGFR ≤ 30 mL/min/1.72 m^2^, due to changes in renal clearance, and also their normoglycemic effect is attenuated as the GFR decreases [6]. Barreto et al. [7] conducted a study to evaluate the pharmacokinetics and safety of dapagliflozin in patients with ESKD undergoing conventional HD regimens compared with age- and sex-matched T2DM controls with normal renal function. The maximum concentration of dapagliflozin was 117 ng/mL and 97.6 ng/mL in patients on HD and in patients with normal renal function, respectively, while the corresponding accumulation ratios were 26.7% and 9.5%. They did not report any serious adverse events (AEs) in either group. In conclusion, the use of dapagliflozin in patients with ESKD in HD was well tolerated, slightly dialyzable, and safe in terms of pharmacokinetics and pharmacodynamics. However, the existing evidence supporting the premise that SGLT-2 inhibitors could be equally effective in reducing cardiovascular events, mortality, and their safety in patients with ESKD on RRT is mainly based on data from post hoc analyses of RCT, experimental studies, or case reports. A post hoc analysis of the CREDENCE study (Canagliflozin and Renal Outcomes in Type 2 Diabetes and Nephropathy) demonstrated a slowing of CKD progression even in patients with advanced diabetic nephropathy [1,8]. Subsequently, during the follow-up period of the DAPA-CKD trial, 2.4 years, 68/2152 and 99/2152 participants in the dapagliflozin and placebo groups, respectively, required the initiation of dialysis. Of these, 25/68 (37%) in the dapagliflozin group and 41/99 (41%) in the placebo group continued the medication [2]. A post hoc analysis was conducted to examine serious AEs among participants who initiated dialysis and continued with the study medication, without finding statistically significant differences in terms of the safety profile [9]. The recent EMPA-KIDNEY (Empagliflozin in Patients with Chronic Kidney Disease) study demonstrated the efficacy of empagliflozin in reducing the risk of the primary composite outcome of renal disease progression or cardiovascular death in patients with CKD, mainly by slowing its progression [3]. Finally, Cao et al. [10] performed a systemic review and meta-analysis to evaluate the effects of SGLT-2 inhibitors in patients with ACKD (eGFR: 15–30 mL/min per 1.73 m^2^). Six RCTs with 2167 participants were included in the quantitative analyses. Among patients with ACKD, SGLT-2 inhibitors reduced the risks of primary kidney and cardiovascular outcomes and attenuated the progressive decrease in eGFR compared with a placebo, with no evidence of additional safety concerns. These observed benefits may support continuing the use of SGLT-2 inhibitors in patients with ACKD before initiating dialysis or kidney transplantation. Currently, the indication for the use of SGLT-2 inhibitors is limited to eGFR values above 20 mL/min/1.73 m^2^. Thus, the recent Kidney Disease: Improving Global Outcomes (KDIGO) guideline for managing CKD considers it safe to continue or even initiate an SGLT-2 inhibitor when eGFR is below 20 mL/min/1.73 m^2^ and continue its use until the initiation of RRT [11].

Moreover, the available clinical data on the use of SGLT-2 inhibitors in PD patients are scarce. Expanding the use of these drugs for their cardio-renoprotective effects in PD patients would be an important and timely step. SGLT-2 inhibitors can offer benefits beyond glycemic control and cardio-renal protection in PD patients, such as the better control volume management, preservation of residual kidney function (RKF), and protecting the peritoneal membrane. Recent experimental studies suggest possible favorable effects of SGLT-2 inhibitors on peritoneal membrane transport function, delaying peritoneal fibrosis, improving the ultrafiltration (UF) of water and toxins, and thereby improving the survival rate of peritoneal patients [12,13]. 

The RKF is of great importance in patients on PD and its benefits extend beyond contributing to achieving adequacy goals [14]. Therefore, the preservation of RKF is crucial for the PD technique and patient survival. Strategies to preserve RKF include prioritizing PD as the “peritoneal dialysis first” modality for initiating RRT, using biocompatible peritoneal dialysis solutions, restricting the use of hyperglycemic PD solutions, avoiding volume depletion, and preventing the use of nephrotoxic agents and peritonitis. Pharmacological tools preserving RKF are limited to the use of drugs that block the Renin–Angiotensin–Aldosterone System (RAAS). In this regard, it has been suggested that SGLT-2 inhibitors may play a role in preserving RKF, although this has not yet been rigorously studied in patients on RRT undergoing PD [15]. Therefore, in the present study, we aimed to assess the potential effects and safety of SGLT-2 inhibitors on the preservation of RKF in both diabetic and non-diabetic patients with ESKD undergoing maintenance PD, during an average follow-up period of six months.

## 2. Materials and Methods

**Figure 1:**
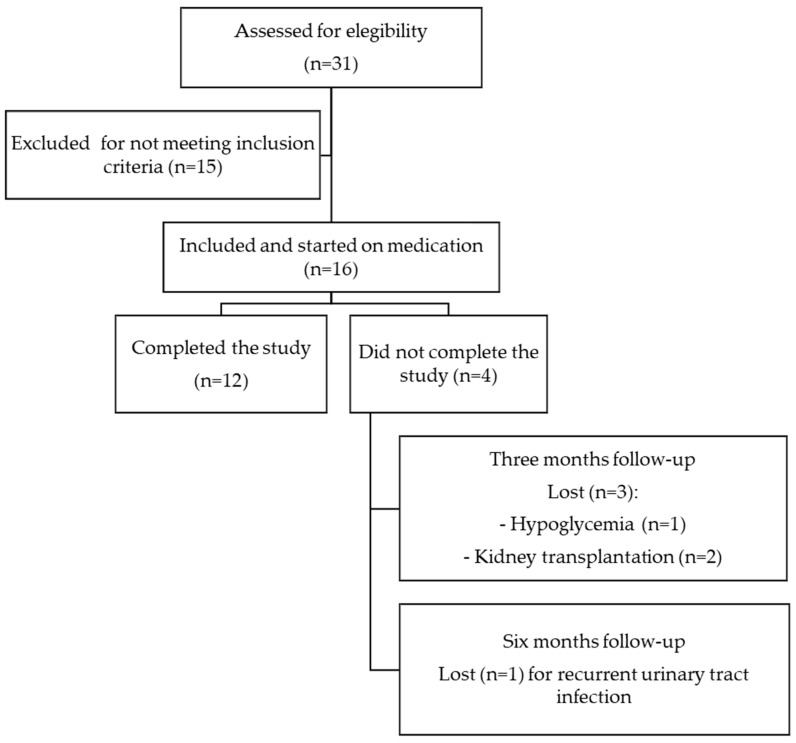
Flowchart of patient inclusion and exclusion criteria.

This is a retrospective observational, single-center study on real-world data; we included patients from the Peritoneal Dialysis Unit of the Hospital General Universitario de Ciudad Real, who started treatment with any type of SGLT-2 inhibitors during the period from December 2022 to December 2023 with an average follow-up period of six months. Although treatment with SGLT-2 inhibitors has not been specifically approved for use in patients undergoing PD, off-label use is based on evidence from clinical trials confirming that the reno-cardiovascular benefits of SGLT-2 inhibitors persist regardless of GFR [1,2,3]. All patients provided informed consent, and the reasons for off-label use of SGLT-2 inhibitors were clearly documented in their medical records. Patients with type 1 diabetes mellitus, recurrent hypoglycemic episodes or diabetic ketoacidosis (DKA), acute or chronic liver disease, history of peritonitis in the past six months, pregnancy, ongoing acute urinary tract infection (UTI) at the time of initiating treatment with SGLT-2 inhibitors, hypersensitivity reactions to SGLT-2 inhibitors, and malignant diseases and those who refused informed consent were not included (Figure 1). The preservation of RKF in patients on PD was defined as the strategy of maintaining the RKF (daily residual urine volume, residual renal clearance rates of urea and creatinine in 24 h urine (KrU and ClCr, respectively)) of patients at an optimal level through PD, with the aim of improving quality of life and patient survival. Therefore, the primary outcome of our study was to evaluate the changes in RKF, assessed as KrU, ClCr, mean of KrU and ClCr, and daily residual urine volume, during a six-month follow-up after the introduction of SGLT-2 inhibitors in prevalent patients undergoing PD. Secondary outcomes included changes in the patients’ blood pressure, mean body weight, 24 h urine protein, parameters of anemia, bone mineral metabolism, electrolyte disturbances, and serum uric acid and determining the presence of AEs after the initiation of treatment with SGLT-2 inhibitors.

We retrospectively collected data on analytical and clinical parameters, KRF (daily residual diuresis urine volume, KrU, and ClCr), peritoneal UF volume, and peritoneal membrane transport function (peritoneal equilibration test [PET], weekly urea Kt/V, and normalized protein catabolic rate [nPCR]) at months 0, 3, and 6, except for standard 4 h PET, which was only performed at the beginning of the study. All participants had been on PD for at least six months. Four patients were on automated peritoneal dialysis (APD) (Baxter’s HomeChoiceClaria) using two changes of 7.5 L of Physioneal 40 Glucose 1.36% and 2.5 L of Nutrineal or 2.5 L of Physioneal 35 Glucose 2.27%, depending on UF needs, with the last fill infusion of 2 L of Extraneal. The remaining patients were on continuous ambulatory peritoneal dialysis (CAPD) with a standard schedule of four two-liter exchanges per day (Fresenius Medical Care with BicaVera and Baxer with Physioneal).

During the follow-up period after the initiation of treatment with SGLT-2 inhibitors, we documented every AE in the patients’ medical records, as well as the discontinuation of SGLT-2 inhibitors. 

The Ethics Committee of Hospital General Universitario de Ciudad Real approved this study (B-307). 

Continuous variables were shown as the mean (standard deviation) or median and interquartile range (IQR), and categorical variables as valid percentages. We compared changes at 6 months as the difference between six months and baseline data. The Kolmogorov–Smirnov test was used to determine whether the data had a normal distribution. Data were analyzed by a Chi-Square test, *t*-test, and repeated one-way ANOVA. A *p*-value < 0.05 was considered statistically significant. The statistical package STATA 16.0 (Stata Statistical Software: Release 16. College Station, TX, USA: Stata Corp LP) was used for the statistical analysis.

## 3. Results

Out of 31 patients in our unit, 16 prevalent patients on PD initiated treatment with SGLT-2 inhibitors (13 with empagliflozin at a dose of 10 mg per day and 3 with dapagliflozin at a dose of 10 mg per day) during the selection process. Of these, 62.5% were male, with a mean age of 67.3 years old. The mean time on the PD until the start of the SGLT-2 inhibitors was 21 months. The baseline demographics and clinical characteristics are shown in Table 1. Baseline peritoneal UF volume was higher in the non-diabetic patient (NDMP) group than in the diabetic patient (DMP) group. However, the residual diuresis volume, KrU, and 24 h proteinuria were higher in the DMP than in the NDMP group. During follow-up, two patients in the NDMP group were transplanted before completing 3 months after the initiation of treatment with SGLT2 inhibitors. Eight patients (50%) had T2DM. Regarding hypoglycemic treatment of T2DM patients, the most common drugs were dipeptidyl peptidase-4 inhibitors (DPP-4i) in four patients (50%), followed by meglitinides in three patients (37.5%), glucagon-like peptide-1 receptor agonists (GLP-1ra) (37.5%) in three patients, and insulin in two patients (25%). Seventy-five percent of the patients were on CAPD modality, and the remaining were on APD. The rest of the baseline characteristics of this study for both groups are presented in Table 1.

**Figure 2:**
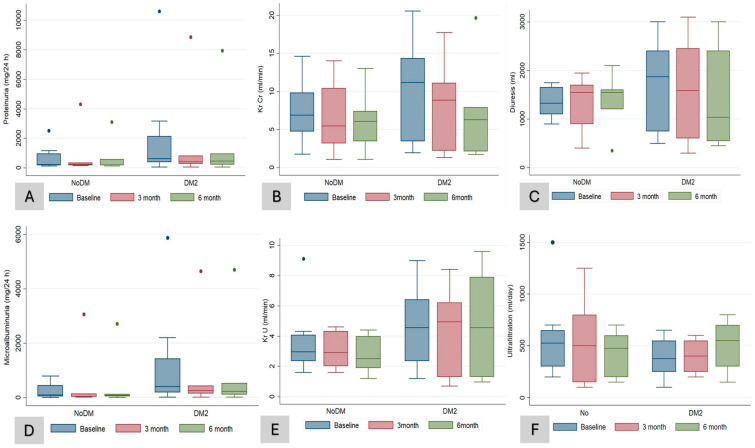
Laboratory data according to groups (DM and NDM patients) during follow-up. Changes in proteinuria in mg/24 h (**A**), KrCr (residual renal clearance rates of creatinine in urine, 24 h) (**B**), diuresis volume (**C**), microalbuminuria (**D**), KrU (residual renal clearance rates of urea in urine, 24 h) (**E**), peritoneal ultrafiltration (**F**) compared at baseline, third, and sixth month after initiation of treatment with SGLT-2 inhibitors in diabetic (DM2) and non-diabetic (NoDM) patients. Box plots show medians with thick line.

After 6 months of treatment, there were no significant (NS) changes in hemoglobin (from 12.2 to 11.8 g/dL, *p* = 0.5), serum uric acid (from 5.2 to 5.4 mg/dL, *p* = 0.5), serum potassium (from 4.8 to 4.6 mmol/L, *p* = 0.1), serum magnesium (from 2 to 2.2 mg/dL, *p* = 0.7), and bicarbonate concentration (from 22.1 to 21.4 mEq/L, *p* = 0.5), nor in other electrolyte or bone mineral metabolism parameters (Table 2). Compared to the blood pressure recording at baseline, we observed a significant drop in the SBP (from 139.8 to 129.7 mmHg, *p* = 0.003) (Table 2). With respect to the RKF after 6 months of treatment with SGLT-2 inhibitors, the patients in both groups preserved RKF (KrU and ClCr) and diuresis (Table 2), with an NS tendency to a reduction in proteinuria (NS). There was an NS increase in the peritoneal UF volume by 50 mL (from 425 to 475 mL/day) (Table 2). When we compared changes at sixth months between DMP and NDMP groups, we did not find significant changes in the parameters of RKF (ClCr, KrU, and diuresis), hemodynamics (SBP and DBP), or the volume of peritoneal UF, except for HbA1c (−0.04 vs. 0%; *p* = 0.04) (Table 3 and Figure 2A–F).

As for AE, only two patients in the DMP groups presented AE; one of them was hypoglycemia (the patient was under hypoglycemic treatment with GLP-1ra and insulin), which was classified as a serious event, of moderate intensity, which required a reduction in insulin doses in addition to the suspension of SGLT-2 inhibitors (empagliflozin), resolving the condition. The other case was asthenia, general malaise, and recurrent urinary tract infection (UTI) (event that had already appeared in previous medical history), which was classified as an event of moderate intensity and required antibiotic and antifungal treatment according to the antibiogram and discontinuation of dapagliflozin.

## 4. Discussion

To our knowledge, we present the first study describing the effects of SGLT-2 inhibitors on RKF preservation in patients with ESKD under PD with or without T2DM. Overall, we observed that the use of SGLT-2 inhibitors during an average six-month follow-up was safe, with few reported AEs consistent with the known safety profile of this class of medication. Second, both diabetic and non-diabetic patients preserved RKF and diuresis during treatment with SGLT-2 inhibitors. Third, there were no changes in peritoneal UF volume in either group during follow-up. Finally, there was a non-significant reduction in albuminuria in our sample at the sixth month; however, it was more pronounced and significant in the DMP group. Lastly, there was a significant reduction in SBP in our sample during treatment with SGLT-2 inhibitors. Due to the potential beneficial pleiotropic effects of SGLT-2 inhibitors, we could consider them an effective and safe therapeutic alternative in patients on PD to reduce albuminuria, preserve KRF, reduce cardiovascular risk, improve health-related quality of life (QoL), and extend the longevity of PD treatment [15].

Currently, most of the available evidence on the use of SGLT-2 inhibitors in PD patients comes from clinical studies with small sample sizes. To date, there are only three published studies using real-world data on the use of SGLT-2 inhibitors in PD patients, which have mainly evaluated effects on changes in peritoneal transport status, peritoneal UF volume, glucose absorption across the peritoneal membrane, residual diuresis volume, and inflammatory markers (serum and peritoneal fluid). Alhwiesh et al. [16] conducted an observational study that included fifty patients with T2DM and ESKD on APD with Baxter’s Home Choice Claria, who were receiving insulin treatment and were prescribed dapagliflozin at 10 mg per day. The main objectives of the study were to evaluate whether treatment with SGLT-2 inhibitors induced changes in the type of peritoneal transport and the volume of UF peritoneal and residual diuresis; additionally, they monitored inflammatory markers such as the erythrocyte sedimentation rate, C-reactive protein, serum ferritin, serum and peritoneal fluid CA-125, and peritoneal fluid effluent polymorphonuclear leucocytes. Although there were no changes in the type of peritoneal transport, at six months of initiating treatment with dapagliflozin, the authors observed a decrease in fasting plasma glucose, HbA1c, and the dose of glargine insulin, as well as a decrease in body weight and SBP. Additionally, there was a significant increase in urine output by 229.9 mL/24-h (from 438.5 to 668.4 mL/24-h, *p* < 0.01) and UF by 0.23 L/day (from 0.438 to 0.67 L/day, *p* < 0.01). Dapagliflozin did not affect urinary ClCr or proteinuria, but some hypouricemic effect was evident in this population undergoing PD. Lai et al. [17] reported a case series of four patients, three of them with T2DM, receiving PD who initiated the technique due to acute pulmonary edema (cases 1 and 3), nausea and vomiting (case 2), and hyperkalemia (case 4). The use of dapagliflozin at 5 mg per day increased peritoneal UF volume, and symptoms of volume overload improved. In contrast to what was reported by Alhwiesh [16] and Lai [17], in our sample, we did not observe a significant increase in peritoneal UF at six months of follow-up, except for a slight increase in UF in the DMP group compared to the NDMP group. On the other hand, Hamdan and colleagues [18] performed an interventional study on twenty patients undergoing CAPD (Fresenius Medical Care) with high and high-average peritoneal transport, who underwent a baseline PET, and one month after starting dapagliflozin at 10 mg per day. The primary objectives were to evaluate changes in D4/D0 glucose, peritoneal solute transfer rate measured by D/P creatinine, and peritoneal UF volume after one month of treatment with dapagliflozin at 10 mg per day. No changes were found in the reduction in peritoneal glucose absorption measured by D/D0 glucose and therefore no increase in UF. This study did not show a statistically significant increase in D4/D0 glucose and thus no reduction in glucose absorption through the peritoneal membrane or increase in UF. Unfortunately, in our study, we did not perform serial PET measurements to assess changes in glucose absorption through the peritoneal membrane, as in our clinical practice, we only perform PET studies when the patient experiences an episode of peritonitis to evaluate changes in the peritoneal transport status.

The preservation of RKF in patients on PD is crucial and a priority in PD units, as it is associated with better QoL, lower incidence of cardiovascular complications, and higher survival rates. Loss of RKF in PD may lead to a worse prognosis and a greater need for adjustments in the dialysis regimen [19]. However, pharmacological tools were limited to the use of an angiotensin-converting enzyme (ACE) inhibitor or an angiotensin II receptor blocker (ARB). In this regard, both ramipril and valsartan have been demonstrated to slow the loss of RKF in patients on CAPD [20,21]. SGLT-2 inhibitors have demonstrated renoprotective properties and, through various mechanisms, may preserve RKF, making them a promising therapeutic option for these patients. The mechanisms by which SGLT-2 inhibitors could preserve RKF include reduction in intraglomerular pressure through renal hemodynamic effects and albuminuria, decrease in oxidative stress and inflammation, and attenuation of renal fibrosis [12,22,23,24,25,26]. In our study, all patients preserved RKF (CrCl and KrU) after the introduction of SGLT-2 inhibitors during the six-month follow-up, with no significant differences found between the DMP and NDMP groups. Currently, there are no trials analyzing the role of SGLT-2 inhibitors in the preservation of RKF in patients on PD. Only one retrospective study of seven diabetic patients undergoing incremental HD demonstrated the efficacy and safety of SGLT-2 inhibitors in preserving RKF [27]. It is known that patients on PD commonly experience a gradual decline in RKF, influenced by factors inherent to CKD and the dialysis process. Many of these patients begin PD with some level of RKF, which they gradually lose over time, contributing to the morbidity and mortality of these patients [28]. In the systematic review and meta-analysis by Cao and colleagues, two of the six RCTs of patients with ACKD treated with SGLT-2 inhibitors included in the quantitative analysis demonstrated that SGLT-2 inhibitors slowed the decline in the eGFR slope, with a difference in eGFR between the SGLT-2 inhibitor group and the placebo group of 1.24 mL/min/1.73 m^2^ per year (95% CI: 0.06–2.42, *p* = 0.04) [10]. Recently, Yen et al. [29] published an emulated target trial study to compare the risk of dialysis, cardiovascular events, and death between users and non-users of SGLT-2 inhibitors in patients with T2DM and stage 5 CKD. By applying the sequential target trial emulation principle, 23,854 SGLT-2 inhibitor users and 23,892 non-users were selected from the Taiwan National Health Insurance Research Database (NHIRD) (from 1 May 2016 to 31 October 2021). The study showed that the risk for dialysis, hospitalization for acute myocardial infarction, and heart failure was substantially lower with the use of SGLT-2 inhibitors than without in patients with T2DM and stage 5 CKD. One of the possible mechanisms by which SGLT-2 inhibitors reduce the risk of CKD progression and dialysis initiation is related to glycosuria-induced osmotic diuresis and natriuresis, which cause afferent arterial vasoconstriction, leading to a reduction in intraglomerular pressure and glomerular hyperfiltration. Although urinary glucose excretion and tubuloglomerular feedback are greatly reduced in ESKD patients (eGFR ≤ 15 mL/min/per 1.73 m^2^), SGLT-2 inhibitors still have beneficial effects in reducing the risk of dialysis. This may be related to the pleotropic effects of these drugs.

Patients on PD frequently present a chronic state of volume overload that is associated with adverse cardiovascular events. In this regard, loop diuretics have been shown to be useful in maintaining water balance by increasing the volume of diuresis. However, they have not been shown to slow the loss of RKF [30]. On the other hand, SGLT-2 inhibitors, by promoting natriuresis and glycosuria, reduce extracellular volume, which may be beneficial in the management of volume overload in PD patients. In our case series, we observed a reduced glycosuria and natriuresis effect associated with low eGFR. Our study did not identify a significant increase in diuresis during follow-up in both groups. We observed a slight increase at the third month, followed by stabilization at the sixth month. Importantly, there was no loss of total diuresis volume at the end of the follow-up period compared to the initial values at the beginning of the study in the DMP group, which is important for us to mention. Similar data were found in Lai et al.’s case series, where the authors did not observe an increase in diuresis in four incident patients on PD, possibly due to the decreased glucosuric effect in this population [17]. However, Alhwiesh et al. [16] reported an increase in urine volume by 253.2 mL/24 h (from 445.6 to 698.8 mL/24 h, *p* < 0.01) in 50 diabetic patients treated with APD after the initiation of SGLT-2 inhibitors at 6 months of follow-up.

In addition, SGLT-2 inhibitors exert their renoprotective effects by decreasing the rate of decline in eGFR and reducing albuminuria [1,2,3]. It is important to describe whether these effects disappear as eGFR falls below 15 mL/min per 1.73 m^2^. The large pivotal CREDENCE, DAPA-CKD, and EMPA-KIDNEY RCTs demonstrated that the efficacy of SGLT-2 inhibitors is independent of the presence or absence of T2DM and of the baseline eGFR under study. The EMPA-KIDNEY study revealed a significant reduction in albuminuria with empagliflozin even in patients with lower eGFR. It is important to note that the nephroprotective mechanism related to the reduction in albuminuria of SGLT-2 inhibitors in patients with T2DM and ACKD is explained more by the synergistic effect of an RAAS blockade on efferent glomerular arterioles to reduce intraglomerular pressure than by the rebalancing of tubule glomerular feedback, which induces the vasoconstriction of the afferent arteriole and thus reduces glomerular hyperfiltration. In our study, we observed a non-significant reduction in albuminuria (from 283 to 114.6 mg/24 h, *p* = 0.9) after 6 months of SGLT-2 inhibitor treatment. Similar data were observed in Alhwiesh´s case series, in which there was no significant change in 24 h urinary protein excretion (from 0.632 g to 0.611 g/24 h, *p* = NS) [16].

In our study, we observed a significant reduction in SBP and non-significant reduction in DBP after 12 months of SGLT-2 inhibitor treatment. Similar data were observed in Alhwiesh´s case series, being the SBP decrease from 148 ± 5.2 mmHg to 134 ± 6.5 mmHg (*p* = 0.0431) after 6 months of SGLT-2 inhibitor treatment. The effects of SGLT-2 inhibitors on blood pressure have been extensively reported [1,2,3]. SGLT-2 inhibitors reduce SBP by approximately 3–6 mmHg and DBP by 1–2 mmHg, leading to decreased pulse pressures [31,32,33,34]. These effects are even observed in patients without T2DM [35,36]. Natriuresis, diuresis, plasma volume contraction, mitigation of arterial stiffness, and enhancement in endothelial functions are the mechanisms postulated to explain this effect [37]. Importantly, this reduction in blood pressure is maintained in patients with low eGFR [8,38]. Furthermore, the blood pressure lowering effect in our study could be related to the fact that most of the patients maintained diuresis above 1500 mL/24 h and concomitantly used an RAAS blockade.

Recent studies have suggested that SGLT-2 inhibitors may have a positive impact on anemia in patients with CKD with and without T2DM [39]. These drugs may improve hemoglobin levels and reduce the need for treatment with erythropoiesis-stimulating agents [40]. Initially, the improvement in hemoglobin was attributed to hemoconcentration due to its diuretic effect and the decrease in plasma volume [41]. However, other mechanisms have been postulated that could be associated with the improvement in anemia after the use of these drugs, such as the reduction in inflammation, the improvement in erythropoiesis by decreasing oxidative stress, and the preservation of RKF [42]. The probable erythropoietic effects of SGLT-2 inhibitors are related to the reversal of relative tissue hypoxia in the proximal tubule as a result of decreased Na^+^/K^+^-ATPase pump activity, secondary to decreased sodium reabsorption by the inhibition of SGLT-2. Additionally, the increased sodium delivery to the distal part of the nephron favors the renal medullary hypoxia, stimulating the synthesis of erythropoietin by interstitial fibroblasts at this location [43,44]. However, this hypothesis is not confirmed in the study by Zanchi et al. [35], suggesting that the increase in erythropoietin levels is influenced by the modulation of the hypoxia-inducible factor, rather than a direct effect of renal hypoxia [44]. We did not observe an increase in hemoglobin levels in either group; instead, there was a non-significant decrease at six months after treatment with SGLT-2 inhibitors (from 12.2 to 11.8 g/dL, *p* = 0.5). Other processes are likely involved in explaining this. Additionally, the patients did not present alterations in iron metabolism, nor were there changes related to the reduction in volume overload induced by these drugs. More research is needed to fully understand the role of SGLT-2 inhibitors in the management of anemia in ACKD and to establish definitive clinical guidelines. In our study, we observed a non-significant weight reduction of approximately 2.9 kg without a significant increase in diuresis. The patients in the study by Alhwiesh et al. [16] experienced a significant weight loss of 2.6 kg compared to their weight before starting treatment with SGLT2 inhibitors (from 86.3 to 83.7 kg, *p* = 0.033). The effects of SGLT-2 inhibitors on weight loss have been described in several studies in patients with CKD and T2DM [1,2,3]. In the recent published substudy of EMPA-KIDNEY of 660 patients with CKD, empagliflozin resulted in a sustained reduction in bioimpedance-derived “Fluid Overload” for at least 18 months, irrespective of diabetes status or level of kidney function. These data from this substudy raise the hypothesis that an important determinant of the 0.7 kg weight loss may be due to effects on fluid status [45]. Unfortunately, in our study, we did not collect data on body compartments measured by bioimpedance.

The use of SGLT-2 inhibitors in patients with ACKD may be associated with certain electrolyte abnormalities. One of these is hyponatremia associated with hypovolemia, which occurs due to osmotic diuresis induced by increased glucose excretion in the urine. Additionally, the inhibition of glucose reabsorption in the proximal tubule can lead to increased sodium excretion, contributing to natriuresis. This sodium loss may predispose patients to hyponatremia, especially in combination with dietary sodium restriction or concomitant use of diuretics. The reduction in serum levels is mild and transient [46]. In our study, we did not observe any alterations in serum sodium, chloride, and potassium levels after 6 months of treatment with SGLT-2 inhibitors. SGLT-2 inhibitors in patients with CKD may influence potassium levels due to their mechanism of action. Although some studies have shown that the use of SGLT-2 inhibitors can result in a slight decrease in potassium levels, especially in patients with CKD, it is important to note that these effects are usually modest and rarely reach clinically significant levels of hypokalemia [47,48,49]. Furthermore, small increases in serum magnesium, calcium, and phosphate concentrations have been described in diabetic patients using SGLT-2 inhibitors [24,50]. In the meta-analysis published by Zhang et al. [24], which included nearly 28,000 diabetic patients, an increase in serum magnesium and phosphate was demonstrated. These data do not coincide with our sample, as we did not observe alterations in serum calcium, phosphate, or magnesium at 6 months after the initiation of treatment with SGLT-2 inhibitors. On the other hand, the efficacy of SGLT-2 inhibitors in reducing serum uric acid levels in patients with T2DM and CKD remains unknown. This effect is primarily attributed to the inhibition of SGLT-2 in the proximal tubule, leading to an increased urinary excretion of glucose and uric acid. Several studies have demonstrated a significant decrease in uric acid levels in patients treated with SGLT-2 inhibitors, which may have important clinical implications as hyperuricemia is associated with an increased risk of cardiovascular and renal disease [51]. Zhang et al. [24] evaluated and ranked the effects and safety of various SGLT-2 inhibitors for serum uric acid levels in patients with CKD. The results showed that SGLT-2 inhibitors significantly reduced serum uric acid levels in patients with CKD compared with the placebo group (standardized mean difference (SMD): −0.22; 95% CI: −0.42 to −0.03; Grading of Recommendations, Assessment, Development, and Evaluations (GRADE): low). In our study, we did not observe a significant reduction in serum uric acid levels. Our data did not agree with those of Alhwiesh et al.´s study [16], which observed a significant reduction in serum uric acid (*p* = 0.0341) but failed to detect a correlation between changes in serum uric acid levels and 24 h urinary uric acid excretion. We did not evaluate this last parameter.

Regarding the safety of SGLT-2 inhibitors, RCTs have shown that the use of SGLT-2 inhibitors in diabetic and non-diabetic patients with CKD is generally safe [1,2,3,4]. A systematic review and meta-analysis of thirty RCTs assessed the safety of SGLT-2 inhibitors in CKD patients and observed that the most frequent AEs were genital mycotic infections, UTI, and DKA [52]. Although it is not an AE per se, during the early phase of treatment with SGLT-2 inhibitors, an increase in creatinine, called permissive acute kidney injury, can occur due to their mechanism of action [53]. We observed in our patients a small deterioration of renal function characterized by a slight elevation in serum Cr (from 6.6 to 7.1 mg/dL, *p* = NS) and a drop in eGFR measured by CKD-EPI 2021 (from 8.3 to 7.5 mL/min, *p* = NS), data expected according to the hemodynamic effects of SGLT-2 inhibitors, without impact on RKF. In the absence of previous clinical trials in PD patients, caution and vigilance should be exercised for any AEs that these drugs may develop in this population. DKA is among the few AEs that occurred more frequently with the use of SGLT-2 inhibitors. In patients on PD, the continuous exposure of the peritoneal membrane to glucose-based PD solutions, together with the effect of SGLT-2 inhibitors, may create an environment conducive to metabolic imbalances, contributing to the risk of euglycemic DKA [54]. Importantly, the use of SGLT-2 inhibitors was associated with increased risks of DKA compared to a placebo in the CREDENCE and SCORED (Effect of Sotagliflozin on Cardiovascular and Renal Events in Patients with Type 2 Diabetes and Moderate Renal Impairment Who Are at Cardiovascular Risk) studies, while this was not observed in DAPA-CKD and EMPA-KIDNEY. This is possibly because the risk of DKA with SGLT-2 inhibitors increases in those with T2DM and decreases in those with more severe CKD. On the other hand, it is not clear to date whether the use of SGLT-2 inhibitors in PD patients could generate a protective effect or a risk of fungal peritonitis (in naïve patients or those undergoing treatment for bacterial peritonitis) in this population. In PD, the use of SGLT-2 inhibitors can lead to an increase in glucose levels in the peritoneal cavity (mainly within the first 60 min). This heightened concentration of glucose, combined with glucose-based solutions, could provide a favorable environment for the growth of microorganisms, increasing the risk of peritoneal fungal infections [37]. Conversely, higher serum hepcidin-25 levels are associated with an increased risk of all-cause and infection-related mortality in PD patients. SGLT-2 inhibitors have been associated with a reduction in hepcidin levels and therefore may have a protective role in cases of PD peritonitis [55]. In our study, two patients in the DMP group developed these AEs. One of them presented an episode of hypoglycemia after the initiation of SGLT-2 inhibitors concomitant with the previously prescribed hypoglycemic treatment (GLP-1ra and insulin) and the other patient had repeated episodes of UTI. The SGLT-2 inhibitors were discontinued in both cases. Similar data were found in the meta-analysis of Cao et al. [10] in ACKD patients treated with SGLT-2 inhibitors. However, it is important to mention that our sample was small, and the follow-up period was only 6 months, which was inadequately short.

Glucose, which is the main osmotic agent used in PD, exerts both local and systemic deleterious effects. Locally, in the peritoneum, glucose and glucose degradation products can cause damage and loss of mesothelial cells, neo-angiogenesis, and peritoneal fibrosis. These local morphological changes result in altered peritoneal function, with rapid transport of small molecules and decreased osmotic capacity, ultimately leading to technique failure. Human peritoneal mesothelial cells express facilitated glucose transporters, GLUT1 and GLUT3, and SGLT involved in active glucose transport against a concentration gradient, SGLT-1 and SGLT-2. An overexpression of SGLT-2 has been observed in the mesothelial cells of the peritoneum of patients with a longer time on PD and, especially, in those who develop encapsulating peritoneal sclerosis [56,57]. Considering the presence of SGLT-2 in peritoneal tissue, it has been hypothesized whether the use of SGLT-2 inhibitors could decrease systemic glucose uptake during PD. To this end, experimental studies have been carried out in animal models and humans. Martus et al. [58] conducted an experimental study in Sprague-Dawley rats infused with dialysis fluid containing 1.5% or 4.25% glucose for a 120 min dwell time with or without empagliflozin administration. The objective was to evaluate the effects of SGLT-2 inhibition with empagliflozin on peritoneal solute and water transport. No significant changes in sodium or water transport across the peritoneal barrier were observed. In contrast, Zhou et al. [13] observed that the intraperitoneal administration of empagliflozin caused a reduction in glucose uptake and thus an increase in UF through the peritoneum of rats. In addition, they detected an inhibition of glucose uptake by peritoneal mesothelial cells in a glucose-rich environment following empagliflozin administration. Similar results were obtained in the study of Balzer [12] and Shentu [59], demonstrating that SGLT-2 inhibition in cellular and animal models of PD was associated with decreased peritoneal glucose uptake and subsequent improvement in peritoneal UF. In addition to influencing peritoneal water and solute transport, it has been suggested that the use of SGLT-2 inhibitors may have beneficial effects on the morphological alterations that occur in the peritoneum exposed to high glucose concentrations [12,59,60]. In our study, unfortunately, we did not evaluate the effects of SGLT-2 inhibitors on peritoneal glucose absorption and peritoneal solute and water transport nor did we monitor inflammatory markers in peritoneal fluid.

To date, there are six ongoing studies involving patients with CKD on PD treated with SGLT-2 inhibitors, and their objectives are (1) evaluating the safety and mechanisms by which SGLT-2 inhibitors can preserve RKF; (2) determining if these drugs can reduce peritoneal glucose absorption and, consequently, have an effect on UF; and (3) evaluating the cardio-nephroprotective effects of SGLT-2 inhibitors in the subgroup of patients undergoing RRT. We look forward with optimism to the results of the following studies: The first, Mechanisms and Safety of SGLT2 Inhibition in Peritoneal Dialysis (CANARY study) [NCT05715814], is an open-label single-center study that will include 20 patients with and without T2DM who are on PD and have RKF, defined as a diuresis of at least 250 mL/24 h and a minimum GFR of 2 mL/min/1.73 m^2^. The primary objective of the study is to evaluate the safety and mechanisms of empagliflozin at 25 mg in PD patients with RKF. The second, Effect of Empagliflozin on Peritoneal and Kidney Function in End Stage Renal Disease (EMPA-PD) [NCT05671991], is a randomized, placebo-controlled clinical trial that will include 30 PD patients with residual diuresis > 400 mL/24 h, who will receive empagliflozin at 10 mg. The primary objective of the study will be to determine if empagliflozin can reduce peritoneal glucose absorption in PD patients. The third is the RENAL LIFECYCLE Trial: A Randomized Controlled Clinical Trial to Assess the Effect of Dapagliflozin on Renal and Cardiovascular Outcomes in Patients with Severe CKD [NCT05374291]. It is a double-blind, randomized, placebo-controlled clinical trial that will include 1500 patients belonging to three subgroups: CKD G4-G5, on dialysis with residual diuresis > 500 mL/24 h, and renal transplant patients with GFR ≤ 45 mL/min/1.73 m^2^. The primary endpoint is a composite of all-cause mortality, renal disease, and hospitalization for heart failure in the study population. Patients requiring the initiation of dialysis or patients on dialysis who receive a renal transplant will continue in the trial and this will not be a reason to discontinue the study. The fourth, Reduction of Peritoneal Glucose Uptake with Use of SGLT2 in Humans Undergoing Peritoneal Dialysis Treatment (PRESERVE) [NCT05250752], is a cross-sectional study that will include 10 patients on PD who will receive dapagliflozin at 10 mg per day. The primary objective will be to determine the glucose value in the peritoneal fluid after a four-hour dwell time and to assess the change in peritoneal glucose reabsorption before and after treatment (three-day treatment period). The fifth is the effect of the use of dapagliflozin in diuresis, natriuresis, and the UF and peritoneal elimination of sodium, in patients with refractory heart failure (DAPA-DP study). The sixth is effects of sodium–glucose co-transporter 2 inhibitors on UF in patients with peritoneal dialysis: a protocol for a randomized, double-blind, placebo-controlled, crossover trial (EMPOWERED) [jRCTs051230081]. In this multicenter, crossover study, patients with clinically diagnosed chronic heart failure are eligible regardless of the presence of diabetes if they use at least 3 L/day of glucose-based PD solutions. Participants will be randomized (1:1) to receive empagliflozin at 10 mg daily and then a placebo or vice versa. The study will enroll at least 36 participants. The primary objective will be the change in daily UF volume from baseline to week 8 in each intervention period. Secondary objectives will be to analyze changes in peritoneal fluid biomarkers, RKF, and anemia-related parameters.

Our study has several limitations that must be considered. Firstly, the sample was small and the follow-up time after the initiation of SGLT-2 inhibitor treatment was relatively short. Secondly, all data were retrospectively collected, and it was an observational study. Therefore, the accuracy and reliability of the data may be questioned. Third, we did not collect patient volume status data with either bioimpedance or bedside ultrasound, nor did we collect serial peritoneal transport status data. Finally, the lack of a control group (patients on PD without receiving SGLT-2 inhibitors) to compare all variables with patients on PD is another limitation of our study. However, despite these limitations, our study has several advantages. As far as we are aware, this is the first study that describes the possible beneficial effects of SGLT-2 inhibitors on preservation RKF in patients with ACKD with or without T2DM on PD. Secondly, our study is unique in including a control group (NDMP) to compare the effects of the use of SGLT-2 inhibitors with diabetic patients on PD.

## 5. Conclusions

In patients with ESKD with or without T2DM receiving maintenance PD, the use of SGLT-2 inhibitors in our small sample appears to be safe, as the AEs experienced by study participants were consistent with the currently known safety profile of the product. SGLT-2 inhibitors may exert positive effects in preserving RKF and diuresis, improving blood pressure control, and reducing proteinuria and weight. These findings need to be confirmed with RCTs to demonstrate robust evidence regarding the efficacy and safety of the use of SGLT-2 inhibitors in patients on PD.

## Figures and Tables

**Table 1:** Baseline demographic data of the sample and by subgroups.

	All (N = 16)	NoDM	DM	*p*-Value
		(N = 8)	(N = 8)	
Age—years, mean (SD)	67.3 (10.3)	64.5 (9.9)	70 (10.6)	0.2
Sex—female, n (%)	6 (37.5)	4 (50)	2 (25)	0.3
Dry weight (Kg), mean (SD)	75.7 (18.3)	70.4 (21.3)	81.1 (14.1)	0.3
BMI (kg/m^2^), mean (SD)	28.4 (6.1)	25.73 (6.31)	31.1 (4.9)	0.07
Causes of CKD, n (%)				0.1
Unknown origin	7 (43.8)	2 (25)	5 (62.5)	
Glomerular disease	5 (31.3)	2 (25)	3 (37.5)	
CTIN	1 (6.3)	1 (12.5)	0	
ADPKD	3 (18.8)	3 (37.5)	0	
HbA1c level, n (%)	6.0 (0.9)	5.4 (0.6)	6.6 (0.8)	<0.001
Hypertension, n (%)	16 (100)	16 (100)	16 (100)	
ACE inhibitors/ARB	16 (100)	8 (100)	8 (100)	
ACC	6 (37.5)	3 (37.5)	3 (37.5)	
Loop diuretics	13 (81.3)	6 (75)	7 (87.5)	
MRA	9 (56.3)	1 (12.5)	8 (100)	
Thiazides	6 (37.5)	4 (50)	2 (25)	
Alpha blockers	5 (31.3)	2 (25)	3 (37.5)	
Beta blockers	7 (43.8)	4 (50)	3 (37.55)	
SGLT-2 inhibitors, n (%)				0.5
Empagliflozin	13 (81.2)	7 (87.5)	6 (75)	
Dapagliflozin	3 (18.8)	1 (12.5)	2 (25)	
PD vintage (months), mean (SD)	21.1 (15.0)	13.1 (8.1)	29.1 (16.5)	0.03
Technical peritoneal dialysis, n (%)				
APD	4 (25)	3 (37.5)	1 (12.5)	0.2
CAPD	12 (75)	5 (62.5)	7 (87.5)	
Technical prescription, n (%)				
Icodextrin	10 (62.5)	5 (62.5)	5 (62.5)	1
Peritoneal equilibrium test:				
D/P, mean (SD)	0.76 (0.12)	0.78 (0.11)	0.74 (0.13)	0.9
D/D_0_, mean (SD)	0.29 (0.1)	0.29 (0.09)	0.29 (0.09)	
Peritoneal membrane function classification:				0.4
Low transporter, number (%)	1 (6.3)	0	1 (12.5)	
Average transporter, number (%)	11 (68.8)	5 (62.5)	6 (75)	
High transporter, number (%)	4 (25.0)	3 (37.5)	1 (12.5)	
Ultrafiltration volume (mL/day), median [IQR]	425 [250–600]	525 [300–650]	375 [250–550]	0.3
nPCR, mean (SD)	0.94 (0.16)	0.94 (0.16)	0.94 (0.18)	0.9
Residual diuresis (L), mean (SD)	1.5 (0.7)	1.35 Lt (0.315)	1.69 (0.9)	0.3
KrU (SD), mL/min, mean (SD)	4.1 (2.5)	3.67 (2.36)	4.6 (2.8)	0.5
ClCr (SD), mL/min, mean (SD)		7.45 (4.2)	10.05 (6.6)	
Kt/V week, mean (SD)	2.1 (0.4)	2.1 (0.2)	2.1(0.6)	0.9
24 h proteinuria (g/day), median [IQR]	489.7 [192.6–1128]	229 [172.8–955.3]	622.9 [398.8–2130]	0.3
MAU 24 h urine (mg/day), median [IQR]	283 [65.2–553]	105.6 [47.6–450.0]	408 [188.7–1414.8]	0.2
SBP (mmHg), mean (SD)	139.8 (10.2)	141 (9.9)	138.6 (11.0)	0.7
DBP (mmHg), mean (SD)	71.4 (8.1)	73.6 (7.4)	69.3 (8.6)	0.3
Bicarbonate, mean (SD)	22.1 (2.1)	22.0 (1.5)	22.1 (2.7)	0.9

Data are shown as mean (SD) or median [interquartile range—IQR] or number (percentage). ADPKD: Autosomal dominant polycystic kidney disease; BMI: Body mass index; CKD: Chronic kidney disease; CTIN: Chronic tubulointerstitial nephropathy; KrU: Residual renal clearance rate of urea in urine, 24 h; ClCr: Residual renal clearance rate of creatinine in urine, 24 h; ACC: Ant Calc channel; MAU: Microalbuminuria; nPCR: Normalized protein catabolic rate; HbA1c: Glycosylated hemoglobin; ACEi: Angiotensin-converting enzyme inhibitors; ARB: Angiotensin II receptor blocker; MRA: Mineralocorticoid receptor antagonist; SGLT-2i: Sodium–glucose cotransporter 2 inhibitors; PD: Peritoneal dialysis; APD: Automated peritoneal dialysis; CAPD: Continuous ambulatory peritoneal dialysis; PET: Peritoneal equilibrium test; D/P: Dialysate/plasma creatinine; D/D0: Ratio of dialysate glucose; SBP: Systolic blood pressure; DBP: Diastolic blood pressure.

**Table 2:** Evolution of variables during follow-up.

	Baseline	3 Months	6 Months	*p*-Value ANOVA
N	16	13	12	
Weight (kg), mean (SD)	75.7 (18.3)	73.6 (16.9)	72.8 (16.7)	0.2
Hb (g/dL), mean (SD)	12.2 (1.5)	11.6 (1.6)	11.8 (0.8)	0.5
Cr (g/dL), mean (SD)	6.6 (2.3)	7.2 (2)	7.1 (1.9)	0.9
CKD-EPI 2021 (mL/min/1.73m^2^), mean (SD)	8.3 (3.6)	7.2 (2.9)	7.5 (3.6)	0.8
Urea (mmol/mL), mean (SD)	139.4 (32.9)	135.8 (28)	131.8 (21.2)	0.2
Sodium (mmol/L), mean (SD)	137.1 (3)	136.4 (3.2)	137.1 (1.9)	0.7
Potassium (mmol/L), mean (SD)	4.8 (0.7)	4.4 (0.5)	4.6 (0.6)	0.1
Chlorine (mmol/L), mean (SD)	100.2 (5)	99 (4.2)	99.6 (3.3)	0.8
Calcium (mg/dL), mean (SD)	9.3 (0.8)	9 (0.4)	9.2 (0.5)	0.3
Phosphorus (mg/dL), mean (SD)	4.8 (1.2)	4.5 (1.1)	4.7 (1.1)	0.6
Magnesium (mg/dL), mean (SD)	2 (0.5)	2.2 (0.5)	2.2 (0.5)	0.7
HbA1c (%), mean (SD)	6 (0.9)	6 (0.7)	5.9 (0.6)	0.8
Uric acid (mg/dL), mean (SD)	5.2 (1.4)	5.1 (1.2)	5.4 (1.3)	0.5
Cholesterol (mg/dL), mean (SD)	143.9 (29.5)	148.8 (47.1)	150.2 (47.8)	0.9
Triglycerides (mg/dL), mean (SD)	152.3 (81.5)	163.5 (80)	193.6 (213.9)	0.7
HDL (mg/dL), mean (SD)	50.1 (15.8)	46.9 (14.5)	43.5 (17.8)	0.5
LDL (mg/dL), mean (SD)	71.8 (17.2)	70.2 (40.3)	64.3 (27.2)	0.6
Bilirubin (mg/dL), mean (SD)	0.4 (0.2)	0.4 (0.1)	0.4 (0.2)	0.6
AST (IU/L), mean (SD)	20.9 (11.7)	23.4 (22.1)	20.5 (15.7)	0.5
ALT (IU/L), mean (SD)	24 (15.8)	27.1 (27.3)	20.5 (12.4)	0.3
GGT (IU/L), mean (SD)	28.2 (21.7)	43.7 (55.3)	36.7 (35.5)	0.2
KT/V weekly (Lt), mean (SD)	2.1 (0.4)	2.1 (0.4)	2 (0.4)	0.5
nPCR (g Urea/Kg/d), mean (SD)	0.9 (0.2)	0.8 (0.1)	0.8 (0.1)	0.08
Ultrafiltration PD (mL/day), median [IQR]	425 [250–600]	500 [250–550]	475 [250–700]	0.8
KrU (mL/min), mean (SD)	4.1 (2.5)	3.7 (2.2)	3.8 (2.7)	0.5
Diuresis (mL/day), mean (SD)	1521.9 (698.8)	1448.5 (837.8)	1402.5 (810)	0.8
ClCr (mL/min), mean (SD)	8.7 (5.5)	7.5 (5.2)	6.7 (5.2)	0.2
(ClCr + KrU/2) (mL/min), mean (SD)	6.4 (3.6)	5.6 (3.6)	5.3 (3.8)	0.2
MAU 24 h (mg/24 h), median [IQR]	283 [65.2–553]	139.5 [42.3–300]	114.6 [56.9–428.4]	0.9
Proteinuria (mg/24 h), median [IQR]	489.7 [192.6–1128]	257.4 [180–500]	328.6 [179–765.4]	0.5
SBP (mmHg), mean (SD)	139.8 (10.2)	129.5 (5.2)	129.7 (7.0)	0.003
DBP (mmHg), mean (SD)	71.4 (8.1)	68.2 (6.8)	71.8(5.9)	0.06
Bicarbonate (mEq/L), mean (SD)	22.1 (2.1)	21.8 (1.3)	21.4 (0.8)	0.5

Data are shown as mean (standard deviation) or median [interquartile range—IQR] or number (percentage). Hb: Hemoglobin; Cr: Creatinine; CKD-EPI: Chronic Kidney Disease Epidemiology Collaboration 2021; HbA1c: Glycosylated hemoglobin; HDL: High-density lipoprotein cholesterol; LDL: Low-density protein cholesterol; AST: Aspartate aminotransferase; ALT: Alanine transaminase; GGT: Gamma-glutamyl transferase; nPCR: Normalized protein catabolic rate; KrU: Residual renal clearance rate of urea in urine, 24 h; ClCr: Residual renal clearance rate of creatinine in urine, 24 h; ClCr + KrU/2: Half sum of ClCr and KrU; MAU: Microalbuminuria; PD: Peritoneal dialysis; SBP: Systolic blood pressure; DBP; Diastolic blood pressure; L: Liters.

**Table 3:** Comparisons of different laboratory indexes between DMG and NDMG patients at sixth month.

	NoDM	DM	*p*-Value
	(N = 6)	(N = 6)	
KrU (mL/min), median [IQR]	−0.1 [−0.4 to 0.2]	0.1 [0–0.6]	0.5
ClCr (mL/min), median [IQR]	−1.1 [−1.6 to −0.7]	−0.3 [−3.1 to 2.1]	0.5
Diuresis (mL), median [IQR]	0 [−150 to 200]	−125 [−600 to 130]	0.5
Ultrafiltration PD (mL/day), median [IQR]	25 [−50 to 50]	50 [0–200]	0.5
HbA1c (%), median [IQR]	0 [−0.07 to 0]	−0.04 [−0.5 to −0.3]	0.04
Proteinuria (mg/24 h), median [IQR]	−15.3 [−40 to 440]	−266.3 [−2209.2 to −10.2]	0.2
MAU (mg/24 h), median [IQR]	−26.9 [−20 to 28.9]	−189 [−1172.6 to −79]	0.05
SBP (mmHg), median [IQR]	−15 [−20 to −7]	−5 [−8 to 2]	0.1
DBP (mmHg), median [IQR]	51 [51–51]	64 [53–68]	0.07
nPCR (g Urea/Kg/d), median [IQR]	−0.1 [−0.31 to 0.01]	−0.05 [−0.3 to 0.1]	0.5
KT/V weekly (L), median [IQR]	0 [−0.02 to 0]	−0.05 [−0.1 to 0]	0.3
Dry weight (kg), median [IQR]	−0.8 [−2.2 to 0.4]	−2.4 [−3.6 to −1.1]	0.1

Data are shown as mean (standard deviation) or median [interquartile range—IQR] or number (percentage). KrU: Residual renal clearance rate of urea in urine, 24 h; ClCr: Residual renal clearance rate of creatinine in urine, 24 h; PD: Peritoneal dialysis; HbA1c: Glycosylated hemoglobin; MAU: Microalbuminuria; SBP: Systolic blood pressure; DBP: Diastolic blood pressure; nPCR: Normalized protein catabolic rate; L: Liters.

## Data Availability

No new data were created or analyzed in this study. The data used to support the findings of this study are available from the corresponding author on request (contact J.C.D.L.F., josedelaflor81@yahoo.com or jflomer@mde.es). We confirm that all the figures and tables are the original work of this manuscript’s authors. All have been created by the authors of this manuscript, have not been adapted from other authors, and do not present an online link.
